# Key features of palliative care service delivery to Indigenous peoples in Australia, New Zealand, Canada and the United States: a comprehensive review

**DOI:** 10.1186/s12904-018-0325-1

**Published:** 2018-05-08

**Authors:** Shaouli Shahid, Emma V. Taylor, Shelley Cheetham, John A. Woods, Samar M. Aoun, Sandra C. Thompson

**Affiliations:** 1 Centre for Aboriginal Studies (CAS), Curtin University, Kent Street, Bentley, WA 6102 Australia; 2 Western Australian Centre for Rural Health (WACRH), School of Population and Global Health, The University of Western Australia, Geraldton, WA 6530 Australia; 3 School of Nursing, Midwifery and Paramedicine, Curtin University, Kent Street, Perth, WA 6102 Australia; 4 Palliative Care Unit, School of Psychology and Public Health, La Trobe University, Melbourne, 3086 Australia; 5 Institute for Health Research, Notre Dame University, Fremantle, WA 6160 Australia

**Keywords:** American native continental ancestry group, Oceanic ancestry group, Aboriginal, Indigenous, Palliative care, Terminal care, End-of-life care, Hospice care, Model of care

## Abstract

**Background:**

Indigenous peoples in developed countries have reduced life expectancies, particularly from chronic diseases. The lack of access to and take up of palliative care services of Indigenous peoples is an ongoing concern.

**Objectives:**

To examine and learn from published studies on provision of culturally safe palliative care service delivery to Indigenous people in Australia, New Zealand (NZ), Canada and the United States of America (USA); and to compare Indigenous peoples’ preferences, needs, opportunities and barriers to palliative care.

**Methods:**

A comprehensive search of multiple databases was undertaken. Articles were included if they were published in English from 2000 onwards and related to palliative care service delivery for Indigenous populations; papers could use quantitative or qualitative approaches. Common themes were identified using thematic synthesis. Studies were evaluated using Daly’s hierarchy of evidence-for-practice in qualitative research.

**Results:**

Of 522 articles screened, 39 were eligible for inclusion. Despite diversity in Indigenous peoples’ experiences across countries, some commonalities were noted in the preferences for palliative care of Indigenous people: to die close to or at home; involvement of family; and the integration of cultural practices. Barriers identified included inaccessibility, affordability, lack of awareness of services, perceptions of palliative care, and inappropriate services. Identified models attempted to address these gaps by adopting the following strategies: community engagement and ownership; flexibility in approach; continuing education and training; a whole-of-service approach; and local partnerships among multiple agencies. Better engagement with Indigenous clients, an increase in number of palliative care patients, improved outcomes, and understanding about palliative care by patients and their families were identified as positive achievements.

**Conclusions:**

The results provide a comprehensive overview of identified effective practices with regards to palliative care delivered to Indigenous populations to guide future program developments in this field. Further research is required to explore the palliative care needs and experiences of Indigenous people living in urban areas.

**Electronic supplementary material:**

The online version of this article (10.1186/s12904-018-0325-1) contains supplementary material, which is available to authorized users.

## Background

Palliative care services aim to improve quality of life (QoL) among patients with life-threatening illnesses and their families [1]. These services provide relief from pain and other distressing symptoms, incorporate psychological and spiritual aspects of patients’ end-of-life (EOL) needs [1], and can support terminally ill patients to die at or close to home [2]. Referral to palliative care early in the course of illness is important for optimal QoL, and also reduces unnecessary hospitalisations and use of health-care services [3]. Growing evidence confirms financial savings associated with palliative care [4]. In 2014, the first ever resolution to integrate hospice and palliative care services into national health services for all people was endorsed by the World Health Assembly [5]. Since then, palliative care has been explicitly recognised under human rights to health. The World Health Organisation (WHO) has endorsed the importance of palliative care to be provided in accordance with the principles of universal health coverage: all people, irrespective of income, disease type or age, should have access to a nationally determined set of basic health services, including palliative care [1]. It has been reinforced that palliative care should be provided through person-centred and integrated health services that pay special attention to the specific needs and preferences of individuals, especially through primary health care and community/home-based care. It is hoped that this endorsement will promote international action to reduce barriers to the accessibility and availability of palliative care.

In developed countries, palliative care now warrants attention as a priority for Indigenous people (the term ‘Indigenous’ refers to First Nation peoples or original inhabitants prior to colonisation in Australia, Canada, NZ and the USA), given their disproportionate burden from chronic diseases and higher mortality rates compared with non-Indigenous people [6]. However, Indigenous populations are among those least likely to receive adequate services [7]. Palliative care service data suggest low rates of utilisation by Indigenous people [8, 9], who often experience multiple episodes of acute hospitalisation for life-limiting conditions [10]. Lack of access to acceptable and appropriate palliative care services among Indigenous populations is a major concern [11]. Furthermore, there are ‘profound cultural dissonances’ [12, 13] between Indigenous and non-Indigenous beliefs in relation to death, disease management, health and health care. Therefore, ensuring cultural respect and sensitivity is of central importance for effective health care delivery to Indigenous peoples [14]. Although priorities have been set, service providers and policy-makers face considerable uncertainty over ways to provide appropriate palliative care to Indigenous peoples, and seek research-based insights to guide practice [14].

The objective of this review was to learn from experiences and inform ways to improve palliative care service delivery for the Indigenous peoples of Australia, Canada, NZ, and the USA, recognising that the Indigenous peoples of these four developed countries share similar histories of colonisation and marginalisation. We aimed to highlight what is known of the needs and preferences of Indigenous patients at the EOL, any barriers to quality care at this time, but primarily the focus was to identify the key features of specific models of care and innovative strategies developed to address these needs, preferences and barriers. We adopted the Agency for Clinical Innovation’s definition of Model of Care (MOC) which is broadly defined as ‘the way health services are delivered. It outlines best practice care and services for a person or population group or patient cohort as they progress through the stages of a condition, injury or event. It aims to ensure people get the right care, at the right time, by the right team and in the right place’ [15] p.3. Innovation in service delivery has been defined as a ‘novel set of behaviours, routines, and ways of working that are directed at improving health outcomes, administrative efficiency, cost effectiveness, or users’ experience, and that are implemented by planned and coordinated actions’ [16] p.582. We combined these two concepts and defined the innovative model of care in EOL for Indigenous populations as: a novel set of behaviours, activities, approaches, initiatives, ways of working that are directed at improving access to, take up and quality of palliative care for Indigenous peoples. This definition was used to identify the key strategies and/ or services that have been applied to deliver EOL care among the Indigenous populations.

## Methods

The review of studies was conducted in accordance with the principles of the Preferred Reporting Items for Systematic Review and Meta-Analysis (PRISMA) statement [17], with the aim of minimising methodological bias, and to ensure accurate and consistent reporting of the review. However, unlike conventional systematic reviews, which are often restricted to specific forms of evidence that are inadequate to explain complex social phenomena, we aimed to synthesise all forms of evidence, including qualitative and quantitative data, an approach similar to that taken by Dixon-Woods (2006) [18].

### Search strategy

The search was from the year 2000 and was conducted in November 2016 across the following databases: PubMed, CINAHL, Embase, PsycInfo, InfoRMIT, Global Health, ScienceDirect, Web of Science and Scopus. The key concepts of ‘palliative care’ and ‘Indigenous’ were searched using a combination of prescribed subject headings and free text keywords. (The search string is presented in Additional file 1: Appendix 1.)

### Screening process: Inclusion and exclusion criteria

**Fig. 1:**
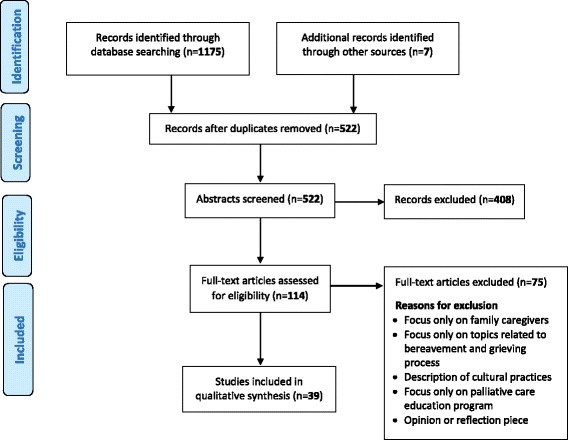
Search strategy and screening process

The screening process is illustrated below using the PRISMA Flow Diagram (Fig. 1). Three authors (SS, ET and JW) independently screened titles and abstracts of publications identified in the search in relation to predetermined inclusion criteria: (i) publication in English language; (ii) publication from the year 2000 onwards (selected to ensure that studies were relatively current); (ii) peer-reviewed articles or full-conference papers; (iv) related to palliative care; and (v) based on findings from Australia, Canada, New Zealand, or the USA.

Four authors (SS, ET, SC and JW) independently reviewed the full text of articles, and conferred in pairs to confirm whether articles met the selection criteria. Any remaining uncertainties were discussed and resolved by the entire team. A publication was excluded following full text examination if it: (i) included no findings on the provision of palliative care to Indigenous patients (such as articles on the delivery of rural-based palliative care without Indigenous-specific findings); (ii) included findings only on advance care planning and EOL care within aged-care services; (iii) had a primary focus on cultural or ceremonial EOL practices; iv) focused on pre and post bereavements rather than on the patients care (covering topics such as the grieving process, bereavement rituals or support); (v) described palliative care education programs, or (vi) was an opinion or reflection piece without research findings.

Multiple papers from the same authors reporting on the same study population were included only if different findings were reported.

### Quality assessment through grading

The methodological quality of the selected publications was assessed using Daly’s hierarchy of evidence-for-practice in qualitative research [Level I: Generalizable Studies; Level II: Conceptual Studies; Level III: Descriptive Studies; Level IV: Single Case Study] [19]. Only 17 key articles that described and explored different models of care were included for grading. Although the use of such tools helps to assess the quality of evidence, it was difficult to grade the included published studies using the traditional taxonomies for levels of evidence [20]. Most publications were descriptive studies (evidence level III) as per Daly’s hierarchy of evidence-for-practice, and many reported findings from program and/or project evaluations where a participatory action research approach was used in designing and developing the program. The heterogeneity in data collection and reporting of findings made it difficult to rate these articles. No article was excluded on the grounds of not meeting a quality standard as our focus was to review papers relevant to the topic, rather than particular study types that met strict methodological standards [18].

### Data extraction

A data extraction pro-forma was developed in Microsoft Excel to assist identifying the details of the study design, aims, sample, study context, analytical framework and key findings, including needs, preferences, barriers, opportunities, and also critical elements of approaches/ initiatives/ models of palliative care service delivery to Indigenous peoples. Data extraction was performed by the authors (SS, ET and SC), initially individually, then working in pairs to confirm the themes. Thematic synthesis [21] was used to analyse the findings. Due to the large volume of data, two clusters of studies were separated: one cluster focused mainly on the barriers, needs and preferences for Indigenous palliative care, and the other one on models of care. Findings related to the needs, preferences, barriers and opportunities have been summarised in tables according to similarity of themes. Themes relating to the primary objective of this review (the models of care) have been inductively derived, interpreted and presented [18].

## Results

Once duplicates (*n* = 660) had been discarded, 515 potentially relevant articles were identified through our search, and an additional 7 through citation snowballing. Of the 522 publications, 408 were excluded during title and abstract screening. Full text review of the remaining 114 publications eliminated a further 75 that did not meet the inclusion criteria, leaving 39 articles to be included in the systematic review.

### Overview of included articles

The 39 papers included were from Australia (9), New Zealand (10), Canada (8), and the USA (12). In 11 studies, the study population consisted of both Indigenous peoples and clinical staff (both Indigenous and non-Indigenous), 17 studies included Indigenous peoples only and three comprised service providers only (both Indigenous and non-Indigenous). Four studies comprised diverse ethnic groups including Indigenous peoples, and five studies did not specifically mention the study population as two of them were literature reviews, and three described specific models for Indigenous populations. The Indigenous populations studied included Aboriginal and Torres Strait Islander Australians, Māori and Samoans, Canadian First Nations (including Cree, Saulteaux/ Anishinaabe, and Lakota/Dakota), Métis, Alaska Natives (including Tlingit/Haida, Yup’ik Eskimo, Inupiaq, Athabascan, Aleut and Alutiiq/Sugpiaq), Native Americans (including Pueblo, Navajo, Hopi and Zuni), and Native Hawaiians.

Most articles (*n* = 33 [85%]) focused on the provision of general palliative care with no terminal condition specified, three focused on palliative care for cancer patients, and one on palliative care for patients with a chronic disease other than cancer. Most studies (29) used qualitative methods only, five were based on mixed methods, two were quantitative, two were literature reviews and one described a palliative care model.

**Fig. 2:**
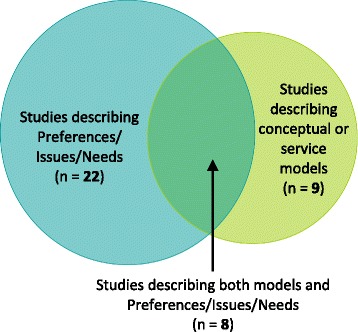
Venn diagram showing clustering of 39 studies meeting review inclusion criteria

The majority of the articles (30) contributed to findings about the palliative care needs and preferences of Indigenous people and the barriers they face in this context. Only 17 papers described actual approaches (presented in Table 4) to making palliative care more accessible to Indigenous peoples, either through the development of a model for delivering palliative care to Indigenous people (conceptual model) or a description of a palliative care service that had been implemented for Indigenous people (service model). These two categories were not mutually exclusive, as eight articles had components relevant to both (Fig. 2).

The first section of the results presents an overview and describes the preferences, barriers and needs of Indigenous people at the EOL identified from the literature. Five main themes on models of care were extracted, with sub-themes identified and supported by several exemplar statements. These will be presented in the final section.

### Needs, preferences and barriers

Tables 1, 2 and 3 present a summary of the key needs, preferences and barriers of Indigenous populations in relation to EOL care.

**Table 1:** Needs of Indigenous populations at the end-of-life

Needs		Australia	Canada	NZ	USA	No. of articles
Collaboration	Community Engagement	X [14, 28]	X [22]	X [24, 26]	X [6, 23, 25, 27]	9
	Family Engagement	X [14, 31]	X [32–34]	X [24, 30]	X [25, 27, 29]	10
	Health Care Provider Collaboration	X [28]	None identified	None identified	X [25]	2
Service Delivery	Funding	X [14, 37]	None identified	None identified	X [6, 23, 25]	5
	Communication	X [14, 31, 37]	X [33, 34, 42]	X [24, 26, 65]	X [6, 23]	11
	Policy Change	X [37]	X [32, 34, 35]	X [26, 65]	X [6, 23, 25, 27]	10
	Staff	X [14, 56]	X [22, 33]	X [24, 26, 36, 65]	X [2, 25, 27, 29, 39]	13
	Built Environment	X [14, 37]	X [22, 33, 35]	X [24, 26, 36, 41]	None identified	9
	Service Delivery, Provision of Care, Capacity of Care	X [14, 28, 31, 40]	X [32, 33, 66]	X [26, 30, 36, 41]	X [2, 6, 25, 27, 29]	16
	Cultural & Spiritual	X [14, 28, 37]	X [22, 32–35, 42, 66]	X [26]	X [25, 27]	13
Education & Training	Training for Health Care Providers	X [14]	X [22, 32, 35]	X [24, 26]	X [2, 6, 23, 25, 39]	11
	Education for Patient, Family and Community	X [40]	X [32]	X [24, 30, 36]	X [6, 27, 38, 39]	9

**Table 2:** Preferences of Indigenous populations at the end-of-life

Preferences		Australia	Canada	NZ	USA	No. of articles
Family and Community	Community Support	None identified	X [32, 35]	X [11]	X [2, 29, 39]	6
	Presence of Families	X [40]	X [32]	X [9, 11, 24, 26]	X [27, 29]	8
	Families Involved in Decision-making	X [14, 31]	X [42]	X [24, 26, 30]	X [27]	7
	Families Involved in Care	X [14]	X [32]	X [9, 11, 26, 41]	X [23, 29, 39]	9
Spiritual and Cultural	Die at Home	X [14, 31, 40]	X [32, 33, 42]	X [11, 26, 41]	X [2, 23, 27, 29, 39, 43]	15
	Ceremonies	X [14, 40]	X [32, 33, 35, 42]	X [26]	X [29, 43]	9
	Language	X [14]	None identified	X [26]	X [39]	3
	Spiritual	X [28, 31, 40]	X [32, 35, 42]	X [41]	X [27]	8
	Pass on Knowledge	X [40]	X [32]	None identified	X [2]	3
Service Delivery	Staff	X [14, 28]	X [32, 42]	X [9]	X [27]	6

**Table 3:** Barriers of access for Indigenous populations at the end-of-life

Issues		Australia	Canada	NZ	USA	No of articles
Accessibility to services	Challenges in Rural and Remote Areas	X [14, 28, 31, 40]	X [32, 42, 66]	X [26, 65]	X [2, 23, 25, 29, 39]	14
	Affordability	X [14]	X [66]	X [11, 24]	X [2, 29, 39]	6
	Lack of Awareness and Knowledge	X [14, 28]	X [66]	X [11, 24, 36, 65]	X [2, 25, 29, 39]	11
Service Delivery	Lack of Funding and Resources	None Identified	X [22, 32, 42]	X [65]	X [2, 6, 23, 25, 39]	9
	Health Service Provider Perceptions	X [28]	None Identified	X [24]	X [43]	3
	Policy	None Identified	None Identified	None Identified	X [2, 6, 23, 25]	4
	Services not Culturally Appropriate	X [14, 40]	X [32, 35]	X [24, 30]	X [29]	7
	Built Environment	X [40]	X [35]	X [26, 65]	None Identified	4
	Staffing Issues	X [14, 28, 40, 67]	X [32, 42, 66]	None Identified	X [2, 23, 25, 29, 39]	12
	Communication	X [14, 28, 31, 67]	X [35, 42, 66]	X [24]	X [6, 25]	11
Cultural Influences	Indigenous Perceptions of Palliative Care	X [14, 40]	X [32]	X [11, 24, 26, 36, 41]	X [25]	10
	Family Conflicts	X [28]	X [32, 33]	X [11, 30]	None Identified	5
	Death Issues	X [28]	None Identified	X [30]	X [29, 39]	4
	Historical, Cultural and Social Context	X [14, 28, 67]	X [22, 42]	X [24, 26]	X [6]	8

#### Needs

Throughout the literature, the need to collaborate and to engage meaningfully with communities [6, 14, 22–28] and families [14, 24, 25, 29–34] before designing and implementing any program was highlighted as a fundamental prerequisite for progress. Most of the studies were conducted in rural or remote locations where EOL care provisions were often not well-developed or well-understood. Better communication, commitment around EOL care at the policy level, staff capacity building, and improved physical environment and access to services were identified as key service delivery needs.

Furthermore, the need for more education and training for both the Indigenous communities and the health care staff in palliative care was identified repeatedly [2, 6, 14, 22–27, 30, 32, 35–40]. Health service providers (HSPs) need to be committed and spend sufficient time to gain the confidence of Indigenous patients and their families.

#### Preferences

There was a strong preference for living with family and within the community at EOL [23, 29, 31, 33, 35–37, 39, 41–43]. Family members generally wanted to be with their loved ones and to fulfil their wishes, including finding ways of enabling care and support to die at home. EOL care was regarded as a process that should involve the entire family, with several studies reporting that families should be at the centre of any decision-making process [24, 26, 27, 30, 31, 44]. Community and/or extended family members gathering was regarded as significant and part of the process for how a dying person is prepared for death [35].

The importance of dying at home or being cared for at home was a common theme for Indigenous people across the four countries [2, 26, 29, 31, 32, 39–41, 43, 44]. Special cultural ceremonies and rituals practiced at the EOL were regarded as important. Dying people benefit from both Western physicians and traditional healers. Elders in one particular study suggested that non-Indigenous HSPs should ask for assistance from ‘elders, priests, spiritual leaders, women who are very strong in their medicine’ (p. 11) if they are unsure of what to do [35]. Thus, in the intercultural space, both worldviews should be respected. Being able to make these connections with families, kin, communities, and land was reported as helping people gain energy and in turn facilitating a strong spirit and peace of mind.

Open and honest communication from physicians, physicians respecting patients’ choices [32], ‘compassion with kindness’ in attitudes [35] and having access to Indigenous staff [42, 44] were expressed as preferences. It was reported in one Canadian study that ‘offering foods that bring comfort to the dying person may be more spiritually and emotionally healing than restrictive diets’ (p.11) [35].

#### Barriers

As expected, distance and affordability of services (along with other indirect expenses related to treatment such as leaving families behind to travel) were identified as key barriers to access palliative care services [6, 14, 22, 24, 26, 42, 45]. Other important service delivery issues included staffing, lack of funding and resources in the sector, and poor availability of culturally appropriate services [6, 14, 22, 24, 26, 42, 45].

Differences between Western medical models and Indigenous cultures in understandings of and priorities for EOL care pose a major issue within the health care setting. Disrespectful and racist treatment by HSPs, and difficulty in regards to communicating EOL care issues to Indigenous patients and families, were also identified as barriers [35, 46, 47]. Hospital policies restricting extended family members from gathering around a dying Indigenous person, or practicing prayers and ceremonies at EOL were highlighted [35]. More training and education for HSPs in order for them to work effectively with Indigenous people who are dying [35], and the employment of more Indigenous staff in the health sector, were seen as possible solutions to these service delivery issues. Misinformation and misunderstandings regarding palliative care and hospice services were also documented among Indigenous people [14, 24, 26], with providing an adequate and continuing health literacy program in the community seen as a requirement for making progress in better EOL care.

### Innovations and models of care

A range of models and innovative service delivery strategies, for delivering EOL care for Indigenous communities across the four countries, were identified from the literature (Table 4). The most comprehensive conceptual model was developed in Australia by McGrath and colleagues [12]. They outlined seven key principles for Indigenous palliative care service delivery: equity (equal access); autonomy/empowerment (respecting patients’ choices); trust (acknowledgement and consideration of the historical context of colonisation and its impact on the lives of Indigenous people and empathy while providing care); humane (non-judgemental care with a focus on quality of life and choice for patients and their families); seamless care (collaboration of a multidisciplinary team of health professionals and community-based organisations, working together across the continuum of care); emphasis on living (rather than on dying), and cultural respect (respect towards cultural practices and beliefs, culturally-based lifestyle) [12].

**Table 4:** Summary of the Key Articles that describe the Models of Care

Author(s), Year, Country, Location	Types of Services	Study Population	Methodology	Models	Critical Elements	Outcomes/ Indicators of Success	Daly’s Hierarchy of Evidence
Braun et al. (2012), USA (mainly rural)	Multiple settings but mainly linking communities and hospitals	Poor and underserved communities including American Indians and Alaska Natives (AI/ANs)	Program analysis using the ‘continuum of cancer care’ and the ‘five A’s of quality care’ frameworks	Patient Navigation (PN) Model	• Early introduction to PC • Focus on the whole cancer continuum • Personal features of patient navigators, such as, capacity to learn about cancer, track cancer services, communicate with professionals, know when and where to refer clients for help, cultural brokers or interpreters for their clients. • Training and orientation to navigators • Continuing education • Flexible approach	Cancer patients have a better quality of life and longer survival when they receive PC concurrently with treatment Cancer PN programs should collect data to track key PN outcomes	Level III
Byock et al. (2006), USA (urban and rural)	Multiple settings, including nursing homes, dialysis clinics, inner city public health and safety net systems and prisons	AI/AN African Americans Medically underserved populations in the city Paediatric patients Mental health patients	Mixed methods evaluation of 22 different projects	Integrated Health Service Delivery (IHSD)	• Community needs assessmentStable institution • Clinician endorsement • Peer-to-peer teaching. • Partnership between the funding bodies and administrators • Successful partnerships, co-ownership and collaboration among academic medical centre, local providers and the community [rural] • Successful projects established working partnerships with local city, country or federal programs [city settings] • Patients and families accepted the delivery model • Established Quality Improvement • Techniques and Routine data collection • PC embedded in cancer care • Creative, careful realignment of existing health system resources • Availability of outpatient PC • Community outreach to raise awareness • On-the-job training • Formal psycho-social and spiritual care QoL assessment tools used to uncover domains of patient or family-reported QoL	Evaluation results are positive: Practicality: Feasibility and Acceptability - 20 of 22 projects were sustained beyond the conclusion of the Grant project - Acceptable to clinicians, administrations, payers, patients and families Access: Availability and use of services - Days for palliative care patients enhanced than national average- Developed partnership with local hospices and with local public health systems to reach to ‘hard-to-reach’ people - Advance Care Planning - Over half of the projects provided education to patients and families Quality: standards, protocols and quality of care - Symptom protocols measured - Regular data collection proved difficult - Good outpatient PC prevented or managed crises that would otherwise require hospitalisation Financial impact: Health care utilization and costs - Costs did not increase - Total health care costs were moderately reduced - Creative, careful realignment of existing health system resources can improve service delivery Ongoing evaluations Project led culture change within the organisations Interest increased in pain management and the social needs of all patients	Level III
DeCourtney et al. (2003), USA (remote)	Decentralised home visiting service	Alaskan Native Villages	Qual Focus groups	Decentralised model	• Community input and engagement • Education and training for Community Health Aides/Practitioners • Multiple referral pathways onto program • Home Health Nurse visits patient • Volunteer coordinator determines support needed • Doctor visits during scheduled village visits 4/5 times/year • Hospital provides out of hours telephone support • Volunteer village youths receive training, help with chores, record traditional knowledge in journal • Integrate all health care and social service resources • Flexible, innovative, patient	• More successful than expected • More patients than anticipated • Patients thrived in home environment and lived longer than expected • Formal evaluation: • - Percentage of home deaths increased from 33% in 1997 to 77% in 2001. - Big increase in number of patients with DNR orders - Caregivers were glad as family member remained in village - AN Health Consortium and “investigating possibility of expanding program to other parts of Alaska”	Level III
Fernandes et al. (2010), USA (mixed)	Kokua Kalihi Valley, a Federally qualified health centre. Offering home based palliative care.	91 HBPC clients enrolled, 46 adult patients	Mixed A prospective design. Data collected upon admission then every month afterwards. Different measures included. A caregiver satisfaction survey & telephone interviews.	Home Based Palliative Care Service Model	• Multidisciplinary team delivers medical care, assesses caregivers for stress & burnout, provides patient & family education • Community partnerships • Routine home visits scheduled every 2–3 months • Bilingual case managers were key to building trust • Local partnerships with universities, churches • Counselling provided • Monthly caregiver support groups • Medical insurance was provided by the physician and psychologist • Family based decision-making • The health centre also serves as a PC clinical rotation for nursing, medical and law schools	• This model has been evaluated • Significant reduction in acute care admissions. • The most utilised support service was case management • High caregiver satisfaction rates • Patients reported significant improvements in wellbeing • The program demonstrated the ability to stabilize the care of seriously and terminally ill patients at home, minimize the pain and anxiety for most clients, improve advance care planning, reduce hospitalisations, and increase appropriate use of community resources	Level I
Finke et al. (2004), USA (rural)		AI/AN	Qual Focus group discussions Interviews	Integrated Health Service Delivery Model	• Collaboration among local health services, communities and university Culturally appropriate materials developed • PC training for clinical staff • Respect and consistency regarding cultural beliefs on death • Strong administrative and management support • Community consultation and needs identified • Local tribal leadership led program • Tribal cultural and spiritual consultation • Distinct PC home health chart • Interdisciplinary team meetings • Coordination with the Zuni EMS • Skilled nursing care • Telephone consultation • Home visits • Adopted policies and procedures	• Development of stakeholder support • Self sustainable	Level III
Kitzes et al. (2004), USA (rural/ remote)	AI/AN Health Care System (IHS facilities)	Secondary data analysis/ 114 Medical Record Review	Mixed methods Medical Record Review and Semi-structured interview	Integrated Service Model in health service settings	• The first IHS Area policy on Palliative Care and Pain Management • Space for traditional ceremonies; • Hospital had an “open door policy” regarding traditional healing; • Spiritual care and cultural practices; • Accommodated families’ desires; • Individualise care; • Not make assumptions about preferences; • Pain Management was developed; • A new version of the IHS patient contact form developed • Policies made available to IHS Elder • Innovative PC programs established involving multiple agencies	This itself was an evaluation paper of one Indian Health Service	Level IV
Kitzes et al. (2003), USA (rural/ remote)	AI/AN Health Care System	Case Studies	Description of multiple initiatives	Service Model	• Cross-trained Home Health Agency employees provided EOL care services, rather than a separate hospice staff. • Medical oncologist provides physician support • “High touch, low tech” program designed • Before start of the program, great effort was ensured to make the services culturally appropriate (medical anthropologist worked with the development team; FGDs conducted to enhance understanding) • Home-based PC and staffed by family and village members. • Nurse’s availability on “on-call” basisInterdisciplinary team discussion	• Evaluation was conducted in some health services • There has been a 500% growth in chronic care patients and a 350% growth in the HHA patients	Level III
Mann et al. (2004), NZ (urban)	Mixed medical/ surgical Intensive Care Unit (ICU)	17 ICU patients (14 NZ Maori, 2 Cook Is Maori, 1 Samoan)	Mixed methods Medical Record Review and discussions with family and health professionals		• Maori patients led • Nurses are experienced, confident, close relationship with family • Palliation plan in place • Multidisciplinary approach • Support from GPs, district nurses, • Hospice Service • Bereavement team – available 24/7 • Approach families of all Maori and Samoan ICU patients facing death • Transport patient home to die • Explain families the process • Patient transported home by 2 ICU nurses, all treatment ceases, pain medication provided • If death is not imminent, PC provided by district nurses, GPs and Hospice	All families reported this as a positive experience	Level III
Slater, et al., (2015), NZ (urban)	Hospice	17 participants	Maori-centered, qualitative research 17 semi-structured, face-to-face interviews with patients, and family members and service providers were undertaken	Hospice-based care	• Importance of building relationships with families, communities and primary health care providers • Building networks with Maori providers, traditional healers • Maori staff partnered with hospice nurse (collaborative model) • Work with volunteer services • Helpful staff • 24 h service • Worked as respite care • Working with family • Accommodating and supportive for large family gathering • Spiritual support provided	• Positive experiences reported • Patients and family members felt more confident with regard to communication • Further needs for improvements explored and documented	Level III
Cottle et al. (2013), NZ (urban)	Hospice	1 woman of Maori and Samoan heritage	Qualitative Single person case study	Whare Tapa Wha Model of Maori health	• Organisational changes occurred to ensure collectivist approach to care • Community engagement and ownership • Support from Maori elders • Coordination between multiple-agencies to deal with the complex case • Multi-systemic and wraparound care • Partnership between cultural community and health care professionals • Clear and regular communication between all parties • A “one size fits all” MOC does not work • Support from hospice – staff time, physical space, management support, nursing clinic • Hui (weekly local gathering) created conditions for significant change to services	• Hui created conditions for significant change to hospice services: • Nursing clinic held during hui meetings • Hui volunteers attended initial assessment • Hui volunteers raised awareness in community • Increased use of hospice by Maori and Pacific people • Availability of and access to palliative care for patients can improve QOL	Level IV
Fruch, et al., (2016), Canada (urban)	Community-based palliative care	Canadian Aboriginal people	Process described	Palliative Shared Care Outreach Team	• Haudenosaunee traditional teachings • Community-based Project Advisory Committee led • Local and regional palliative care partners led implementation; partnership with researchers • Vision was to deliver compassionate, coordinated and comprehensive EOL • Community capacity development • Locally initiated and driven • Dedication, leadership and commitment from key community members and local healthcare providers • Bottom up approach • Built on existing resources and infrastructure • Community had required infrastructure, i.e., health services and providers • Shared vision for change • Effective collaboration among community healthcare providers and members • Community members feeling empowered • 24/7 Palliative Shared Care Outreach Team providing medical, spiritual and cultural support	• Palliative care guidelines and client care pathways are in effect • Increased home deaths as opposed to hospital or hospice deaths • Number of referrals increased • Increased access to palliative care education • Mentorship opportunity for local healthcare providers • Incorporation of traditional teachings to support clients and staff dealing with death and dying	Level III
Kelly et al. (2009), Canada (rural)	Hospital, Palliative Care Service	10 bereaved Aboriginal family members	Qual Semi structured interviews	Service model in hospital setting	• Services extended to visiting family • Interpreter service • Empower patient to decide place of death • Infrastructure • Involvement of all hospital staff • Spiritual care • Participant experiences considered to make changes in services, cultural practices and physical surroundings	Yes – ongoing qualitative evaluation	Level III
St Pierre-Hansen et al. (2010), Canada (rural/ remote)	Rural Health Centre	3 different baseline studies: Community Consultation: 50 elders FN PC Study: Qualitative study: 10 participants whose family members received PC	Qual Patient survey, Group discussion In-depth interviews	Service Model	• Leadership and governance based on the cultural values and beliefs • Active community engagement in decision-making and planning stage • Minimise communication barriers and provide support services • Cultural training to staff • Infrastructural/ environmental transformation occurred • Traditional healing and cultural needs incorporated • Elders provided patient support • Interpreters trained as certified medical interpreters • Planned telephone follow-up of bereaved families • Two-day cultural orientation and conflict-resolution training program	• Some form of evaluationMore planned - telephone follow-up of bereaved families • Interpreter availability increased from 50 h/month to 250 h/month - patient satisfaction increased	Level III
McGrath (2010), AUS (remote)		72 participants – patients (10), carers (19), AHWs (11), health professionals (30), interpreters (2)	Qualitative Open-ended qualitative interviews	The Living Model for Aboriginal Palliative Care Service Delivery – Conceptual Model	• Considered patients within the context of the extended family • Cultural safety, Community participation, Personal advocacy, Choice, Empowerment • Understand/support/ respect cultural grief practices • Focus on staying at home • Education – consumer and professional • Facilitate family meetings • Service availability in the communities • Address psychosocial and practical problems • Effective communication • Use of Indigenous workers • Provision of respite • Carer and escort support • Advocacy for resources and infrastructure	Not evaluated	Level I
McGrath et al. (2006), AUS (remote)		72 participants	Qualitative Open-ended qualitative interviews	Indigenous Palliative Care Service Delivery Conceptual Model	1) Equity 2) Autonomy and Empowerment 3) The Importance of Trust 4) Humane, Non-judgmental Care 5) Seamless Care 6) Emphasis on Living 7) Cultural Respect	Not evaluated	Level I
McGrath et al. (2009), AUS (remote)		72 participants	Qualitative Open-ended qualitative interviews	Service model	• Generic features of palliative care: - 24 h access to palliative care - Focus on living - Respect for choice and autonomy - Patient advocacy - Support to patients, families - Patience and compassion - Multidisciplinary skill - Expert advice - Interagency cooperation - Seamless care - Dedicated professionalism - Carer upskilling - Provision of respite care • Rural and remote specific factors: - Practical assistance (support [oxygen] and organisational [Meals on Wheels]) - Flexible and creative approach to solve some practical issues - Health professionals visit communities • Cultural respect - Relationship and trust-building - Family and community network - Respect for grieving practices - Physical environment - Use of traditional healer and respect for spiritual practices	Not clear	Level I
Carey, et al., (2016), AUS (remote)	Alice Spring Palliative Care Service, NT	Patients accessing the services	Cross-sectional qualitative study/ evaluation study	Day Respite Facility	• Respite care available in the locality • Flexibility of the staff; Staff attitudes • Relationship and friendship with staff • Provision for caring for complex patients, and looking after their clinical, personal needs • Transportation provided • Service was flexible and accommodating	• Qualitative evaluation • Impact has been strongly positive • Therapeutic needs ensured • Client satisfaction • Symptom management, medication compliance, QoL and service coordination – all improved • Act as a ‘safe place’ for isolated and marginalised community members • ED attendances and hospital admissions dropped	Level II

Hands-on, practical, and innovative service delivery models were identified as having adopted diverse strategies to deliver palliative care into communities. The service models included: Patient Navigators Model [48], Outreach Care [11] and Palliative Shared Care Outreach Model [49] and Home-based service [4], Hospice based care [9, 11], and Integrated Health Service Delivery (IHSD) Model [7, 23, 50]. Eleven of the 17 articles that discussed service models were based on rural/remote locations, two covered mixed locations and four were based in an urban context [mostly within health services settings except for Fruch et al. [49].

In most cases, evaluations of these service models have been included in the published literature, with the following positive short-term outcomes reported (Table 4): symptom management, medication adherence and patients’ QoL improved [4, 7, 11, 48, 51, 52]; total health care costs moderately decreased [7]; emergency department (ED) attendances and hospital admissions diminished [52]; service use and patient satisfaction increased [34, 52]; number of deaths at home increased [2, 49]; and families and caregivers reporting positive experiences with the services [4, 9, 51]. Kelly et al., [33] reported there would be ongoing qualitative evaluation. Two remote communities in Australia’s Northern Territory (NT) developed their palliative care services according to McGrath’s ‘Living Model’ (Table 4) [14] although it was unclear whether the model had been evaluated in these settings.

The contexts for implementing these innovations varied: some were in-patient hospital or hospice settings whereas others were in community-based care settings. Common themes and critical elements that have facilitated EOL service delivery to Indigenous populations are discussed below (Table 5).

**Table 5:** Critical elements of models of care in an Indigenous setting identified from the published, peer-reviewed literature

Community Engagement	• Community/ local needs identified • Strong community connection and engagement in decision-making, planning, designing the program/ project • Community leadership
Education & Training: Providers, Support Workers & Carers	• Upskilling staff through training • Providing training and education to community members (peer-to-peer teaching) • Culturally-appropriate resources and materials
Culturally Safe Service Delivery Strategy	• Palliative Care integrated with cancer care (palliative care is not separated rather included within the cancer treatment continuum, Link to an established Program) • A team-based whole-of-service approach (Support from all staff) • Creative, careful realignment of existing health system resource utilisation • Clinician endorsement is critical
Flexible Organisation/ Program Structure	• Sufficient flexible funding • Stable institution • Infrastructure (physical environment, Built Environment, accessibility and availability of services) • Organisational policy • Partnership with local agencies, hospitals, academic institutions, etc.
Patient-centered Care	• Culturally safe care (respect for traditional practices and medicine, respectful of traditional beliefs, providing cultural and spiritual care) • Delivery of Care (Inter-disciplinary care, multidisciplinary team, coordination of care, outreach services/ home visit, interpreter services) • Family involvement in care and decision-making, place of death, home visit, outreach services, provide various forms of support, patient empowerment, compassionate care)
Quality Service Delivery	• Ongoing evaluation • Systematic record-keeping to capture progressive data

#### Community engagement

Effective service models implemented in rural or remote settings demonstrated strong community connection and involvement from the outset [2, 7, 11, 33, 49]. Some had built partnerships with local services, some had involved Elders from the communities in the program design and materials development, others reported that they had explored community palliative care needs before designing the projects [2, 7, 23], while some promoted services that were already well-established in the local communities. One study reported that an eight person Elders’ Council had influenced strategic planning and operations, and that this Council had shaped the program in the locality [34]. Elders also provided patient support by visiting patients in their residences and as interpreters. Kitzes et al. [6] included tribal and community values in planning, and considered the diversified community and their rich history, sacred culture and traditions [6]. Fruch and colleagues [49] also reported traditional philosophies guiding the project development. Cottle et al. [11], in their case analysis in one urban-based hospice service, explained how they adopted the Whare Tapa Wha Model of Maori health (consisting of four dimensions: spiritual, mental, physical, extended family) into their service delivery [11]. They worked with a Hui (weekly local gathering) to make significant changes to that hospice service, including reallocating staff time, rearranging the physical space, and re-orienting the management format. They made efforts to ensure clear and regular communication between all parties. These initiatives increased use of that particular hospice service by Māori and Pacific peoples.

#### Education and training

Providing continuing education and training to upskill HSPs and community stakeholders and family members is an integral part of all community-based program models [2, 7, 11, 23, 48]. Byock and colleagues [7] highlighted the significance of peer-to-peer teaching within their program. The palliative care and primary health care (PHC) partnership model described by DeCourtney et al. [2] provided training to village-based workers to develop a cadre of trained workers and volunteers in each Alaskan Native Village. Specific culturally sensitive program materials were developed and used to educate and train patients, families, staff, and volunteers. Training for navigators is an integral part of the Patient Navigators’ model [48]. McGrath et al. [14] also highlighted the importance of consumers and professionals’ education in their conceptual model. The service models that were implemented in the hospital or health service settings adopted other strategies, such as cultural orientation and conflict-resolution training programs for staff.

#### Culturally safe service delivery strategy

Various service delivery strategies have been identified. Attributes of individual staff were identified as particularly crucial for service delivery models like the Navigator Model, whereas the IHSD Model [7] adopted a whole-of-service approach (team-based palliative care) in which all staff within the service were informed and involved in delivering palliative care to clients. Where palliative care was integrated within the existing services, funds and resources were generally more sustainably shared and allocated [7, 11, 23, 33], and strong support was usually received from administrative and management staff [23]. Clinicians’ endorsement was identified as particularly important to ensuring implementation and continuing delivery of palliative care in health services settings. DeCourtney et al. [2] reported that they did not want to introduce new strategies to deliver palliative care, but instead utilised an existing rural health care delivery model in the Alaskan Native Villages to expand the continuum of care to the EOL setting. They described it as a decentralised model that combined trained volunteers and health care workers in villages, with medical direction from a central urban location and home visits by nurses. They allowed for multiple referral pathways into the program, including by family members. Three studies described decentralised home-based outreach palliative care service models [2, 4, 49] with a large multidisciplinary team including outreach workers, delivering clinical care by making regular home visits every 2–3 months. Bilingual case managers, monthly caregiver support groups and a family-based decision-making process were also part of the service model. DeCourtney et al. [2] stated that doctors visited remote villages 4–5 times per year whereas Fruch et al. [49] described a Palliative Shared Care Outreach team that offered medical, spiritual and cultural care 24/7 to the communities. Carey et al. [52] described a ‘Day Respite Facility’ in the Alice Spring EOL service in the Northern Territory in Australia which had helped reduce emergency attendances and hospital admissions at the end stages of life while addressing therapeutic needs and client satisfaction.

#### Flexible organisation/ program structure

A report on innovative palliative care programs noted that they tended to be more successful when implemented in stable institutions, i.e., those not experiencing severe financial stress or undergoing structural changes [7]. Successful programs were flexible in nature and embraced co-ownership and collaborative partnerships. In rural settings, partnerships occurred between academic medical centres, local providers and the community [23]. In urban settings, established working partnerships operated with local city, county or federal programs. These programs mostly worked with established support services, such as volunteer programs [7].

Organisational level changes were reported in Outreach Care, a non-residential, community-based hospice organisation in New Zealand, when the organisation was required “to move beyond Eurocentric individualism to a more collectivist approach to care” [11]. Outreach Care ensured holistic assessment of patients’ physical, emotional, psychosocial and economic needs; invited local community members to tell the service about their unmet needs; involved multiple agencies to deal with individual cases; and maintained clear and regular communication between all parties [11].

Palliative care service providers sometimes underwent infrastructure refurbishments to make their service more comfortable and accessible to Indigenous patients and their families [14, 33, 34, 53, 54]. Examples included: the construction of a new building with a smudge room (in which the smoke of sacred herbs is used for ceremonial purification), enlarging a common area in order to accommodate large family groups, and ensuring the availability of large patient rooms [11, 52].

#### Patient-centred care

Patient-centred care that is “respectful of and responsive to the preferences, needs and values of patients and consumers” was prioritised in all these service models. The dimensions of patient-centred care are “respect, emotional support, physical comfort, information and communication, continuity and transition, care coordination, involvement of family and carers, and access to care” [55] p.13.

The availability of navigators’ support during EOL phases was successful in ensuring that patients found cancer care understandable, available, accessible, affordable, appropriate, and accountable. Navigators work as cultural brokers and interpreters for their clients, and ensure that the clients are participating fully and actively in care [48]. One navigator noted,

*“when a client is terminal, we work hard to take a neutral position relative to cancer treatment. We provide information and allow them to make their own decisions about continuing chemotherapy and other treatments. If a client starts saying he/she is ‘tired of treatment and pain’ and ‘it’s time to return to God’, we discuss what the client and family want of the future, and provide information about advance directives, palliative care, and hospice.”* [48]

As part of the IHSD Model, implemented through the Promoting Excellence in EOL Care program in the USA, different communities adopted different locally suitable strategies to promote EOL care. The IHSD model introduced new standards and protocols to ensure delivery of core palliative care services: pain and symptom management, psychosocial care, spiritual counselling and support, QoL improvement and continuity of care, value-based care, and life-review [7]. Twenty of the 22 projects were sustained in some form by their home institutions beyond the conclusion of the program funding. DeCourtney et al. [39] described how, as part of the IHSD program, they established a village-focused, culturally sensitive, regionally based physician- and home health nurse-led multi-disciplinary palliative care program in rural Alaska Native communities. The Helping Hands Program provided training to village-based health care providers on palliative care, and these trained health care providers provided at-home care during EOL. This model allowed for multiple referral pathways while helping to decentralise services, by ensuring central technical support from a local health service. When patients were admitted into the program, four steps were followed: 1) individual needs-based assessment; 2) identification of differences in goals between patient and service providers; 3) individual care plan development concordant with community values; and 4) establishment of trust. Patients and family members were pleased with the option to remain at home in familiar surroundings as they neared the EOL. The frequency of nurses’ visits to patients’ homes was increased if the patient’s condition worsened and bereavement support to family members after a patient’s death was also provided.

#### Quality improvement in service delivery

Most of the projects, especially those under the Promoting Excellence in Palliative Care program in the USA [7], used established quality improvement techniques for systematic record-keeping and to monitor and observe program impacts on patient outcomes. Byock et al. [7] described how various projects refined their palliative care service delivery strategies based on feedback from clients and observed changes in outcomes. Clinical data were used in care planning, including the use of QoL assessment tools to highlight domains of patient or family-reported needs and helped to focus therapeutic attention.

## Discussion

This comprehensive review of the literature has identified a variety of key innovative strategies for delivering palliative care to Indigenous communities in Australia, NZ, Canada, and the USA. Preferences, barriers and needs that can influence quality of palliative care for Indigenous patients and their families have also been examined. Despite diversity amongst the included Indigenous communities, similarities in terms of the needs and preferences were observed in the literature. Many of the issues identified are not likely to be very different from those of other people at the EOL; however, HSPs drawing on those that were more unique to Indigenous people might make a big difference to the care for Indigenous people globally. Overwhelmingly the included publications focused on community-based palliative care services. There were very few examples in the literature of culturally safe palliative care delivery within hospital inpatient settings or specifically designed ‘stand-alone’ inpatient palliative care facilities (for example, hospices). This could be because of the preference of Indigenous people to be cared for at home [56] (as identified in this review) or because many Indigenous people do not feel safe in hospitals [57]. Clearly, admission to a specialised palliative care inpatient facility may be required for brief episodes of care (i.e. respite, acute symptom stabilisation).

Key preferences identified are: family and community involvement; dying at home; provision for cultural and spiritual ceremonies within service settings; open and honest communication from health professionals; respectful treatment by HSPs, and availability of Indigenous staff. Indigenous people expressed a strong preference to spend time with families and communities at EOL. Families are pivotal to the wellbeing of dying Indigenous patients [11, 24, 41]. In congruence with that need, service models have been developed to ensure that families are included in clinical decision-making. Efforts have been made to build relationships with family members and carers, to promote respect for family caregivers’ roles, and to facilitate death at home when appropriate. Reconnection with the land before death is frequently highlighted as a strong preference for Indigenous people across the four countries. It was observed that some innovative services endeavour to bring people back to their ‘homeland’ to die. However, additional staffing of personal support workers, outreach community workers, nurses and case managers are required to facilitate the choice of dying on the homeland, and for some people, at home. When quality palliative care enables people to die at home, community members are more willing to engage in the care process [49]. Hospices in New Zealand have tailored their services to meet the needs of Māori patients by increasing flexibility, partnering Māori hospice staff with both non-Indigenous staff and primary health care providers, working closely with families, creating physical space for large families to visit, and regular communication between multiple agencies [11]. Community leadership of EOL program development in rural and remote Indigenous communities facilitates education and training of support workers, in turn creating employment opportunities.

The major barriers that restrict access to culturally appropriate palliative care services include: distance from and cost of services; a paucity of culturally safe service environments; disrespectful treatment by HSPs, poor communication, and differences in understanding of and priorities at the EOL between HSPs and Indigenous people. We have identified six critical elements within the identified models that attempted to deliver culturally sensitive palliative care services to Indigenous populations and address the above-mentioned preferences and needs: community engagement, education and training, culturally safe service delivery strategy, flexible organisation/ program structure, patient-centred care, and quality service delivery.

Key models and innovative service strategies for improving Indigenous access to palliative care services must ensure a culturally safe environment for Indigenous families by employing appropriate Indigenous health workers within the services, providing compulsory cultural awareness programs for all staff, and creating opportunities for community awareness-raising [57]. Where these preferences were addressed adequately, improved quality of care was achieved in terms of access to services, client satisfaction, symptom management, and corresponding declining ED and hospital admissions. Engagement and partnership of palliative care programs with existing local health services has been a key to success, especially in rural and remote settings. Likewise, within the urban context and for inpatient settings, strong relationships and regular communication with primary health care providers can play a pivotal role in expediting referrals to palliative care services, and in endorsing the value of palliative care facilities to patients, families and other health services [49]. Such actions can facilitate the trust-building process between patients, family members and service providers, and alleviate fears around palliative care services. However, further research is required to explore the palliative care needs and experiences of Indigenous people living in urban areas.

Health partnerships at national, provincial and regional levels are important in promoting culturally safe palliative care service delivery for Indigenous populations. Despite ‘palliative care’ being identified as a ‘priority area’ at all levels of care and to the whole population in international policy documents, major barriers such as, lack of public and professional awareness of the benefits of palliative care, workforce shortages, lack of infrastructure and care delivery models and an inadequate evidence base have made EOL care inaccessible to many people [58]. Moreover, in the developed world, palliative care has become synonymous with service provision, rather than with its original purpose, as an ethos and approach to care. Under this ethos, palliative care begins at the time of the diagnosis, however in practice, care has tended to be provided only the last months and weeks of life, due to limited resources. Therefore, many population and disease groups lack access to specialist palliative care. From the studies synthesised in this comprehensive review, Indigenous peoples seem to be supported during the terminal illness and end of life in ways that fit with the key principles of a palliative approach to care. A palliative approach to caring emphasises patient- and family-centred care that focuses on the person and not just the disease, the importance of therapeutic relationships between care providers and the patient and family and clear communication throughout the illness trajectory about goals of care, comfort measures, and needs and wishes [59, 60]. Therefore, the way forward, is to upskill primary care professionals in indigenous communities in the principles and practice of a palliative approach to care for a more sustainable model of care. Although not Indigenous-specific, one such upskilling program has been successful in the field of Motor Neurone Disease [61].

More recently, in the wealthy nations, including Australia, NZ and Canada, government funding is being allocated strategically [58]. In Australia, increasing government interest in all aspects of reducing disparities in Indigenous health and closing service gaps has been evident in Close the Gap campaigns and other programs. Government contracts for a broad spectrum of health and education services now contain a clause specifically requiring providers to address Indigenous issues. There have also been many Indigenous-specific EOL initiatives developed by non-governmental organisations, i.e., Palliative Care Australia (Program of Experience in the Palliative Approach [PEPA] [62]), the Palliative Care Outcomes Collaboration [PCOC]) [63] and Cancer Australia.

In this context, it is hoped that evidence of practical, context-specific frameworks or models of care will contribute to enhancing the understanding of particular needs of different population groups, which in turn will ensure universal coverage of appropriate delivery of palliative care to all population groups.

## Conclusion

Health equity is an important goal and includes efforts to ensure equitable access to quality treatment, resources and appropriate support. However, Indigenous people have been underrepresented in palliative care services and this is an important issue for attention. This review has highlighted the key features of culturally safe service delivery that have been reported to be working well in the Indigenous palliative care context. “‘Good care’ is defined by those receiving the care, and not those who provide it” [64]. A flexible approach, adaptability to the context and ‘buy-in’ from local communities are reported to be some of the essential features of successful service models to deliver palliative care services to Indigenous populations and the literature emphasises that a ‘one size fits all’ approach is not appropriate [11]. This flexibility must incorporate family involvement in decision-making [4] and extend to the referral process, such that family members are able to refer patients to specialist palliative care services [2, 23]. McGrath et al. [14], reiterated that, “a static model … [should not] be imposed on services or communities but rather a living, flexible model is required to assist with service delivery and health policy” [p59]. Flexibility in these settings also augments Indigenous representation and retention within the health workforce [9].

## Additional file

Additional file 1: Multi-database search string. (DOCX 17 kb)

## Abbreviations

AI/ANs: American Indians and Alaska Natives
ED: Emergency Department
EoL: End-of-Life
FGDs: Focus Group Discussion
GP: General Practitioners
IHS: Indian Health Service
HSPs: Health Service Providers
ICU: Intensive Care Unit
IHSD: Integrated Health Service Delivery
MOL: Model of Care
NT: Northern Territory
NZ: New Zealand
PC: Palliative Care
PCOC: The Palliative Care Outcomes Collaboration
PEPA: Program of Experience in the Palliative Approach
PHC: Primary Health Care
PN: Patient Navigation
PRISMA: Preferred Reporting Items for Systematic Review and Meta-Analysis
QoL: Quality of Life
USA: The United States of America

## References

[1] World Health Organisation. Palliative Care. Fact sheet N°402. WHO. 2015. http://www.who.int/mediacentre/factsheets/fs402/en/. Accessed 04 Apr 2018.

[2] DeCourtney CA, Jones K, Merriman MP, Heavener N, Branch PK (2003). Establishing a culturally sensitive palliative care program in rural Alaska Native American communities. J Palliat Med..

[3] Meier D (2011). Increased access to palliative care and hospice services. Opportunities to improve value in health care. Milbank Q..

[4] Fernandes R, Braun KL, Ozawa J, Compton M, Guzman C, Somogyi-Zalud E (2010). Home-based palliative care services for underserved populations. J Palliat Med.

[5] National Hospice and Palliative Care Organisation. Ground-breaking palliative care resolution is adopted at World Health Assembly in Geneva. Media release. 2014. http://www.globalpartnersincare.org/news/ground-breaking-palliative-care-resolution-adopted-world-health-assembly-geneva. Accessed 03 Apr 2018.

[6] Kitzes J, Berger L (2004). End-of-life issues for American Indians/Alaska Natives: insights from one Indian Health Service area. J Palliat Med..

[7] Byock I, Twohig J, Merriman M, Collins K (2006). Promoting excellence in end-of-life care: a report on innovative models of palliative care. J Palliat Med.

[8] Hampton M, Baydala A, Drost C, McKay-McNabb K (2009). Bridging conventional Western health care practices with traditional Aboriginal approaches to end of life care: a dialogue between Aboriginal families and health care professionals. Can J Nurs Inform..

[9] Slater T, Matheson A, Ellison-Loschmann L, Davies C, Earp R, Gellatly K, Holdaway M (2015). Exploring Maori cancer patients', their families', community and hospice views of hospice care. Int J Palliat Nurs.

[10] Australian Institute of Health and Welfare. Australia’s health 2014. Australia’s health series no. 14. Cat. no. AUS 178. Canberra: AIHW; 2014.

[11] Cottle M, Hughes C, Gremillion H (2013). A community approach to palliative care: embracing Indigenous concepts and practices in a hospice setting. J Syst Ther..

[12] McGrath P, Holewa H (2006). Seven principles for Indigenous palliative care service delivery: research findings from Australia. Austral-Asian J Cancer..

[13] McMichael C, Kirk M, Manderson L, Hoban E, Potts H (2000). Indigenous women's perceptions of breast cancer diagnosis and treatment in Queensland. Aust N Z J Public Health.

[14] McGrath P (2010). The living model: an Australian model for Aboriginal palliative care service delivery with international implications. J Palliat Care..

[15] Agency for Clinical Innovation. Understanding the process to develop a Model of Care–An ACI Framework. Version 1.0. Chatswood: Agency for Clinical Innovation; 2013.

[16] Greenhalgh T, Robert G, MacFarlane F, Bate P, Kyriakidou O (2004). Diffusion of innovations in service organizations: systematic review and recommendations. Milbank Q.

[17] Liberati A, Altman DG, Tetzlaff J, Mulrow C, Gøtzsche PC, Ioannidis JP (2009). The PRISMA statement for reporting systematic reviews and meta-analyses of studies that evaluate healthcare interventions: explanation and elaboration. BMJ.

[18] Dixon-Woods M, Cavers D, Agarwal S, Annandale E, Arthur A, Harvey J (2006). Conducting a critical interpretive synthesis of the literature on access to healthcare by vulnerable groups. BMC Med Res Methodol.

[19] Daly J, Willis K, Small R, Green J, Welch N, Kealy M, Hughes E (2007). A hierchachy of evidence for assessing qualitative health research. J Clin Epidemiol.

[20] Aoun S, Kristjanson L (2005). Evidence in palliative care research: how should it be gathered?. Med J Aust.

[21] Nicholson E, Murphy T, Larkin P, Normand C, Guerin S (2016). Protocol for a thematic synthesis to identify key themes and messages from a palliative care research network. BMC Res Notes.

[22] Castleden H, Crooks VA, Hanlon N, Schuurman N (2010). Providers' perceptions of Aboriginal palliative care in British Columbia's rural interior. Health Soc Care Community..

[23] Finke B, Bowannie T, Kitzes J (2004). Palliative care in the Pueblo of Zuni. J Palliat Med..

[24] Frey R, Gott M, Raphael D, Black S, Teleo-Hope L, Lee H, Wang Z (2013). 'Where do I go from here'? A cultural perspective on challenges to the use of hospice services. Health Soc Care Community.

[25] Isaacson M, Karel B, Varilek BM, Steenstra WJ, Tanis-Heyenga JP, Wagner A (2014). Insights from health care professionals regarding palliative care options on South Dakota reservations. J Transcult Nurs.

[26] Taylor JE, Simmonds S, Earp R, Dip PT. Māori perspectives on hospice care. Divers Equal Health Care. 2014;11:61–70.

[27] Schrader S, Nelson M, Eidsness L (2009). Reflections on end of life. Comparison of American Indian and non-Indian peoples in South Dakota. Am Indian Culture Res J.

[28] Shahid S, Bessarab D, Van Schaik KD, Aoun SM, Thompson SC (2013). Improving palliative care outcomes for Aboriginal Australians: service providers’ perspectives. BMC Palliat Care..

[29] Colclough YY, Brown GM (2014). American Indians experiences of life-threatening illness and end of life. J Hosp Palliat Nurs.

[30] Frey R, Raphael D, Bellamy G, Gott M (2014). Advance care planning for Maori, Pacific and Asian people: the views of New Zealand healthcare professionals. Health Soc Care Community.

[31] Fried O (2003). Palliative care for patients with end-stage renal failure: reflections from Central Australia. Palliat Med.

[32] Hotson KE, Macdonald SM, Martin BD (2004). Understanding death and dying in select First Nations communities in Northern Manitoba: issues of culture and remote service delivery in palliative care. Int J Circumpol Health..

[33] Kelly L, Linkewich B, Cromarty H, St Pierre-Hansen N, Antone I, Giles C. Palliative care of First Nations people: a qualitative study of bereaved family members. Can Fam Physician. 2009;55:394–5. e397. PMC2669014 19366951

[34] St Pierre-Hansen N, Kelly L, Linkewich B, Cromarty H, Walker R (2010). Translating research into practice: developing cross-cultural First Nations palliative care. J Palliat Care..

[35] Hampton M, Baydala A, Bourassa C, McKay-McNabb K, Placsko C, Goodwill K (2010). Completing the circle: elders speak about end-of-life care with Aboriginal families in Canada. J Palliat Care..

[36] Bray Y, Goodyear-Smith F (2013). Patient and family perceptions of hospice services: 'I knew they weren't like hospitals'. J Prim Health Care.

[37] Brooke NJ (2011). Needs of Aboriginal and Torres Strait Islander clients residing in Australian residential aged-care facilities. Aust J Rural Health..

[38] Colquhoun S, Dockery A (2012). The link between Indigenous culture and wellbeing: qualitative evidence for Australian Aboriginal peoples. CLMR Discussion Paper Series 2012/01.

[39] Decourtney CA, Branch PK, Morgan KM (2010). Gathering information to develop palliative care programs for Alaska's Aboriginal peoples. J Palliat Care..

[40] Dembinsky M (2014). Exploring Yamatji perceptions and use of palliative care: an ethnographic study. Int J Palliat Nurs.

[41] Bellamy G, Gott M (2013). What are the priorities for developing culturally appropriate palliative and end-of-life care for older people? The views of healthcare staff working in New Zealand. Health Soc Care Community.

[42] Kelly L, Minty A (2007). End-of-life issues for Aboriginal patients: a literature review. Can Fam Physician..

[43] Marr L, Neale D, Wolfe V, Kitzes J (2012). Confronting myths: the Native American experience in an academic inpatient palliative care consultation program. J Palliat Med..

[44] Davidson PM, Jiwa M, DiGiacomo ML, McGrath SJ, Newton PJ, Durey AJ (2013). The experience of lung cancer in Aboriginal and Torres Strait Islander peoples and what it means for policy, service planning and delivery. Aust Health Rev..

[45] Shahid S, Finn L, Bessarab D, Thompson S (2011). 'Nowhere to room ... nobody told them': logistical and cultural impediments to Aboriginal peoples' participation in cancer treatment. Aust Health Rev.

[46] Oetzel J, Simpson M, Berryman K, Iti T, Reddy R (2015). Managing communication tensions and challenges during the end-of-life journey: perspectives of Maori kaumatua and their whanau. Health Commun.

[47] Oetzel JG, Simpson M, Berryman K, Reddy R (2015). Differences in ideal communication behaviours during end-of-life care for Maori carers/patients and palliative care workers. Palliat Med.

[48] Braun KL, Kagawa-Singer M, Holden AEC, Burhansstipanov L, Tran JH, Seals BF (2012). Cancer patient navigator tasks across the cancer care continuum. J Health Care Poor Underserved.

[49] Fruch V, Monture L, Prince H, Kelley ML (2016). Coming home to die: six nations of the Grand River Territory develops community-based palliative care. Int J Indig Health..

[50] Kitzes JA, Domer T (2003). Palliative care: an emerging issue for American Indians and Alaskan Natives. J Pain Palliat Care Pharmacother..

[51] Mann S, Galler D, Williams P, Frost P (2004). Caring for patients and families at the end of life: withdrawal of intensive care in the patient's home. N Z Med J.

[52] Carey TA, Schouten K, Wakerman J, Humphreys JS, Miegel F, Murphy S, Arundell M (2016). Improving the quality of life of palliative and chronic disease patients and carers in remote Australia with the establishment of a day respite facility. BMC Palliat Care.

[53] McGrath P (2006). Exploring Aboriginal peoples' experience of relocation for treatment during end-of-life care. Int J Palliat Nurs..

[54] McGrath PD, Phillips EL (2009). Insights from the Northern Territory on factors that facilitate effective palliative care for Aboriginal peoples. Aust Health Rev..

[55] Australian Commission on Safety and Quality in Healthcare. Patient-centred Care: Improving quality and safety by focusing care on patients and consumers. Discussion Paper. Sydney: ACSQHC; 2010.

[56] McGrath P, Holewa H, Kail-Buckley S (2007). "They should come out here ...": research findings on lack of local palliative care services for Australian Aboriginal people. Am J Hosp Palliat Care.

[57] Australian Commission on Safety and Quality in Healthcare (ACSQHC). Consumer health information needs and preferences: Perspectives of culturally and linguistically diverse and Aboriginal and Torres Strait Islander people. Sydney: Cultural & Indigenous Research Centre Australia; 2017.

[58] Morrison RS. A National Palliative Care Strategy for Canada. J Palliat Med. 2017;20(Suppl 1):S63–75. 10.1089/jpm.2017.0431 PMC5733738 29283876

[59] Kristjanson LJ, Toye C, Dawson S: New dimensions in palliative care: a palliative approach to neurodegenerative diseases and final illness in older people. Med J Aust. 2003; 179(Suppl 6):S41-3. 10.5694/j.1326-5377.2003.tb05578.x 12964937

[60] Stajduhar KI, Tayler C: Taking an “upstream” approach in the care of dying cancer patients: the case for a palliative approach. Can Oncol Nurs J. 2014;24:144-53. 25189052

[61] McConigley R, Aoun S, Kristjanson L, Colyer S, Deas K, O'Connor M (2012). Implementation and evaluation of an education program to guide palliative care for people with motor neurone disease. Palliat Med.

[62] Shahid S, Ekberg S, Holloway M, Jacka C, Yates P, Garvey G, Thompson SC. Experiential learning to increase palliative care competence among the Indigenous workforce: an Australian experience. BMJ Support Palliat Care. 2018. 10.1136/bmjspcare-2016-001296. 10.1136/bmjspcare-2016-001296 PMC6582728 29353253

[63] Eager K, Watters P, Currow DC, Aoun SM, Yates P (2010). The Australian palliative care outcomes collaboration (PCOC) – measuring the quality and outcomes of palliative care on a routine basis. Aust Health Rev.

[64] Ramsden IM (2002). Cultural safety and nursing education in Aotearoa and Te Waipounamu (PhD Thesis).

[65] Lawrenson R, Smyth D, Kara E, Thomson R (2010). Rural general practitioner perspectives of the needs of Maori patients requiring palliative care. N Z Med J..

[66] Prince H, Kelley M (2010). An integrative framework for conducting palliative care research with First Nations communities. J Palliat Care..

[67] McGrath PD, Patton MA, Ogilvie KF, Rayner RD, McGrath ZM, Holewa HA (2007). The case for Aboriginal Health Workers in palliative care. Aust Health Rev..

