# Supplementary material for: Key features of palliative care service delivery to Indigenous peoples in Australia, New Zealand, Canada and the United States: a comprehensive review
Source: BMC Palliat Care. 2018 May 8;17:72. doi: 10.1186/s12904-018-0325-1 (PMC5938813; doi:10.1186/s12904-018-0325-1)
Supplement: Supplementary file 1 — Multi-database search string. (DOCX 17 kb) [file 12904_2018_325_MOESM1_ESM.docx]

**Appendix 1**

**Palliative care service delivery to Indigenous peoples in Australia, New Zealand, Canada and the United States: A systematic review**

**Multi-database search string 04.11.2016**

| **DATABASE** | **REFERENCES RETRIEVED** |
| --- | --- |
| **PubMed** |  |
| **(Palliative Care [mh] OR Terminal Care [mh] OR Hospice and Palliative Care Nursing [mh] OR “palliative care” OR “terminal care” OR “hospice care” OR “end-of-life care” OR “palliative medicine” OR “palliative management” OR “non-curative care” OR “noncurative care” OR “remedial care”) AND (health services, indigenous [mh] OR oceanic ancestry group [mh] OR american native continental ancestry group [mh] OR indigenous OR indigene* OR aborigin* OR “torres strait islander” OR “torres strait islanders” OR maori* OR polynesian* OR “pacific peoples” OR “native american” OR “native americans” OR “american indian” OR “american indians” OR amerind* OR alaskan* OR eskimo* OR “native hawaiian” OR “native hawaiians” OR “first nation” OR “first nations” OR inuit* OR metis OR “native canadian” OR “native canadians” OR “canadian indian” OR “canadian Indians”) AND 2000:2017 [dp]** | **176** |
| **CINAHL-Plus** |  |
| **MH "Terminal Care+" OR MH "Hospice and Palliative Nursing" OR “palliative care” OR “terminal care” OR “hospice care” OR “end-of-life care” OR “palliative medicine” OR “palliative management” OR “non-curative care” OR “noncurative care” OR “remedial care”**  **AND**  **MH "Health Services, Indigenous" OR MH "Indigenous Peoples+" OR MH "Indigenous Health" OR indigenous OR indigene* OR aborigin* OR “torres strait islander” OR “torres strait islanders” OR maori* OR polynesian* OR “pacific peoples” OR “native american” OR “native americans” OR “american indian” OR “american indians” OR amerind* OR alaskan* OR eskimo* OR “native hawaiian” OR “native hawaiians” OR “first nation” OR “first nations” OR inuit* OR metis OR “native canadian” OR “native canadians” OR “canadian indian” OR “canadian Indians”**  **(Date-restricted 2000-2017)** | **186** |
| **Scopus** |  |
| **TITLE-ABS-KEY((palliative PRE/2 care) OR (terminal PRE/2 care) OR (hospice PRE/2 care) OR (palliative PRE/2 medicine) OR (palliative PRE/2 management) OR (non-curative PRE/2 care) OR (noncurative PRE/2 care) OR (remedial PRE/2 care)) AND TITLE-ABS-KEY(indigenous OR indigene* OR aborigin* OR “torres strait islander” OR “torres strait islanders” OR maori* OR polynesian* OR “pacific peoples” OR “native american” OR “native americans” OR “american indian” OR “american indians” OR amerind* OR alaskan* OR eskimo* OR “native Hawaiian” OR “native Hawaiians” OR “first nation” OR “first nations” OR inuit* OR metis OR “native canadian” OR “native canadians” OR “Canadian indian” OR “Canadian Indians”) AND PUBYEAR AFT 1999** | **175** |

| **PsycINFO** |  |
| --- | --- |
| **(exp palliative care/ or exp hospice/ or palliative care.mp. or palliative therapy.mp. or terminal care.mp. or hospice care.mp. or end-of-life care.mp. or palliative medicine.mp. or palliative management.mp. or non-curative care.mp. or noncurative care.mp. or remedial care.mp.) and (exp indigenous populations/ or indigenous.mp. or indigene$.mp. or aborigin$.mp. or torres strait islander$.mp. or maori$.mp. or polynesian$.mp. or pacific people$.mp. or native american$.mp. or american indian$.mp. or amerind$.mp. or alaskan$.mp. or eskimo$.mp. or native hawaiian$.mp. or first nation$.mp. or inuit$.mp. or metis.mp. or native canadian$.mp. or canadian indian$.mp.)**  **limit to yr="2000 - current"** | **81** |
| **EMBASE** |  |
| **(exp palliative therapy/ or exp palliative nursing/ or exp terminal care/ or palliative care.mp. or terminal care.mp. or hospice care.mp. or end-of-life care.mp. or palliative medicine.mp. or palliative management.mp. or non-curative care.mp. or noncurative care.mp. or remedial care.mp.) and (exp aborigine/ or exp american indian/ or exp eskimo/ or exp indigenous people/ or exp maori/ or exp pacific islander/ or indigenous.mp. or indigene$.mp. or aborigin$.mp. or torres strait islander$.mp. or maori$.mp. or polynesian$.mp. or pacific people$.mp. or native american$.mp. or american indian$.mp. or amerind$.mp. or alaskan$.mp. or eskimo$.mp. or native hawaiian$.mp. or first nation$.mp. or inuit$.mp. or metis.mp. or native canadian$.mp. or canadian indian$.mp.)**  **limit to yr="2000 - current"** | **305** |
| **Global Health** |  |
| **(hospice care/ or palliative care or terminal care or hospice care or end-of-life care or palliative medicine or palliative management or non-curative care or noncurative care or remedial care)**  **AND**  **(indigenous people/ or aborigines/ or alaska natives/ or american indians/ or inuit/ or pacific islanders/ or indigenous or indigene$ or aborigin$ or torres strait islander$ or maori$ or polynesian$ or pacific people$ or native american$ or american indian$ or amerind$ or alaskan$ or eskimo$ or native hawaiian$ or first nation$ or inuit$ or metis or native canadian$ or canadian indian$)**  **limit to yr="2000 - current"** | **29** |
| **Web of Science** |  |
| **(palliative NEAR/2 care OR terminal NEAR/2 care OR hospice NEAR/2 care OR end-of-life NEAR/2 care OR palliative NEAR/2 medicine OR palliative NEAR/2 management OR non-curative NEAR/2 care OR noncurative NEAR/2 care OR remedial NEAR/2 care)**  **AND**  **(indigenous OR indigene* OR aborigin* OR “torres strait islander” OR “torres strait islanders” OR maori* OR polynesian* OR “pacific peoples” OR “native american” OR “native americans” OR “american indian” OR “american indians” OR amerind* OR alaskan* OR eskimo* OR “native Hawaiian” OR “native Hawaiians” OR “first nation” OR “first nations” OR inuit* OR metis OR “native canadian” OR “native canadians” OR “Canadian indian” OR “Canadian Indians”)**  **2000-2017** | **189** |

| **ScienceDirect ‘All Science’ – journals only** |  |
| --- | --- |
| **pub-date > 1999**  **and**  **TITLE-ABSTR-KEY("palliative care" OR "terminal care" OR "end-of-life care" OR "hospice care" OR "palliative medicine" OR "palliative management" OR "non-curative care" OR "noncurative care" OR "remedial care")**  **and**  **TITLE-ABSTR-KEY(indigenous OR indigene* OR aborigin* OR "torres strait islander" OR "torres strait islanders" OR maori* OR polynesian* OR "pacific peoples" OR "native american" OR "native americans" OR "american indian" OR "american indians" OR amerind* OR alaskan* OR eskimo* OR "native Hawaiian" OR "native Hawaiians" OR "first nation" OR "first nations" OR inuit* OR metis OR "native canadian" OR "native canadians" OR "Canadian indian" OR "Canadian Indians")** | **8** |
| **Informit – all databases – journals only** |  |
| **(SUBJECT:indigenous OR SUBJECT:indigene* OR SUBJECT:aborigin* OR SUBJECT:"torres strait islander" OR SUBJECT:"torres strait islanders") AND (SUBJECT:"palliative care" OR SUBJECT:"terminal care" OR SUBJECT:"end-of-life care" OR SUBJECT:"hospice care" OR SUBJECT:"palliative medicine" OR SUBJECT:"palliative management" OR SUBJECT:"non-curative care" OR SUBJECT:"noncurative care" OR SUBJECT:"remedial care")**  **2000-2017** | **26** |
| **TOTAL (prior to removal of duplicates)** | **1175** |
